# A Case of Congenital Stricture of the Vagina, Complicating Labor

**Published:** 1855-12

**Authors:** Wm. C. Butler

**Affiliations:** East Avon


ART. III.—*A Case of Congenital Stricture of the Vagina, Complicating Labor.* By Wm. C. Butler, M. D., East Avon.

The limited opportunity for medical research possessed by most country practitioners, leads them, no doubt, into the frequent error of supposing that they have to do with anomalous cases, when, if they were more conversant with medical literature they would find those very cases not only common but trite. An humiliating truth I confess. But truth is said to be often stranger than fiction. Such may be the fact with regard to the following case, and the only inducement for me to offer it you, is the vain imagination that perchance there be one of the numerous readers of your valuable Journal who may be as much in the *fog* as the one who resides in dark and benighted Avon.

A very limited ride in this locality for a few years, has not as yet presented a parallel case. How unfortunate others may have been I know not.

Case. Was called, Dec. 1, 1844, to see Mrs. R., aged 34, in labor with her first child. Learned that she had been in labor thirty-six hours, and that for the last twenty-four hours the pains had been regular, and averaging once in ten minutes. Also learned that she was one of that peculiar class that did not believe in a male accoucheur, and had sternly resolved up to the time of my being sent for, to carry out her prejudices.

On entering the room, I found a short, thick set, strong, muscular woman, who had had her neck, some eight years previous, slipt (unconsciously I suppose) into the hymenial knot, and whether she considered herself as then slipping it out, I know not, but do know that her appearance exhibited strong mental and physical excitement. Pulse 90, full and strong. Face flushed as is frequently the case in the last stage of labor. Countenance anxious and exhibiting much suffering. Still no particular organ suffering from congestion; when free from the pain of labor was quite easy and inclined to rest. Upon making a digital examination, found nothing unusual until about one and a half inches frnm the labia pudendi. I at first supposed that it was the os uteri dilated to the size of the index finger; but upon carrying it still farther up to make out the presentation, found that it was still surrounded by what felt like the cartilaginous rings which compose the trachea. Still farther on I could detect the membranes distended by each pain, and also that some portion of the cranium was presenting. The mobility of the parts would not admit a more correct diagnosis. The os uteri, I had no doubt, was fully dilated, and the only difficulty to the progress of an otherwise natural labor, was this stricture. I could not learn by the mother, who was present, that there ever had been any mechanical or chemical causes which could produce it; or by the patient herself.

Taking her liege lord aside and inquiring minutely into the peculiarly mysterious POWERS and *properties* of coition, the poor soul told me, with tears in his eyes, that he must confess that after eight years of patient, persevering, and unwearied toil, he had been unable to pass the Rubicon; but supposed that it was the end of all *terrestrial* things, as he had one of the fairest of Eve's fair daughters, as well as the embodiment of perfection itself.

After being fully satisfied that the effort of nature was doing nothing toward dilating the stricture, I stated to them the nature of the impediment, prospects, &c., and requested counsel. To this she would not for a moment listen; said she would submit to anything I might wish save having another man around her; and sagely came to the conclusion that she had had two too many already.

Such being the situation of matters at this period, I concluded to open a vein in the arm, and reduce the action of the heart, not in the vain hope that it would relax matters somewhat, but sure that I could, according to Beck, get the full effects of an anodyne to better advantage. Bled her 24 oz., and gave her one gr. sulph. morph. in half a tumbler of water. She soon felt the effects of the opiate without effecting the labor pains.

Upon examining the case still more carefully, I found that the rugous folds of the mucous membrane gave it the appearance, in feel, of the fibrous rings like the trachea, and that the fibrous band was undoubtedly of uniform thickness, and gave me the impression — for it could be but an impression — of being a line in thickness, and two inches in length.

Such, then, was the congenital impediment to an otherwise natural labor. How was it to be overcome at that day, age and stage of labor? Or in other words, how was she to be safely delivered? This was the only query of any importance to me just then.

After looking my thick storehouse carefully over in vain for a precedent or authority, I threw that matter all one side (a slight all, for I could not recall a case nor any authority) and came down to the case itself.

It will be seen at a glance that there were but two horns to the dilemma: 1st. To overcome the stricture; 2d. That formidable operation, the Cæsarean section, which must ever be considered a dernier resort.

Again. If we look at the case with a view of overcoming the stricture, it will be seen at a glance that the knife alone has any properties worthy of consideration at this time.

I said I could not think of any authority that would assist in this dilemma. This I will recall. Prof. Willard Parker, who stands preëminent as a teacher and surgeon, in 1840 gave this rule as the rule of all rules in surgery: "Gentlemen, know well the anatomy of the parts with which you have to do, and be sure that with it you are familiar; then let the first principles of common sense (no mean ingredient you see) dictate you, and my word for it, you will have no cause to regret. Believe me it is worth volumes for your guidance." Unfortunately your humble servant was, and is, destitute of the second ingredient in this important compound. Endeavoring to act upon this principle, I took a long straight bistoury, and with the aid of a grindstone destroyed the cutting edge to within about half an inch of the point, and, then by winding this with a thread, protected it from injuring the soft parts. My impressions were, that if I could divide the stricture into three or four nearly equal parts, the mucous membrane would dilate so that the child could be dissected and delivered, *per vias naturales.*

As she had felt no motion for the last two weeks, and the stethescope did not show any evidence of fœtal circulation, I came to the conclusion that the child had been for some time dead. Strange as it may seem, both these impressions proved to be correct. Placing the patient on her back in the usual position for instrumental labor, I ordered two females to sponge the soft parts as dry as possible up as far as they could, and then making two convenient and beautiful spatules of the index and middle fingers of my fair assistants, had those spatules introduced as far as possible, drawing the vagina forcibly externally. With this assistance I carried the index finger of the left hand to the edge of the stricture with the flat side of the knife upon it. As soon as I felt the fibrous cord-like band, I punctured the mucous membrane, keeping the point of the finger in advance of the point of the knife, carrying it with caution, in the interim of pains, through to the farther side of the stricture, endeavoring to keep the mucous membrane between the finger and the knife. I then turned the cutting edge from the finger and resigned its farther direction to the middle finger and thumb of the same hand. I then introduced the index finger of the right hand into the rectum, and carried it up opposite said point. Fancied that I could in this way tell nearly the depth of the incision, or at least whether the rectum was in any danger. By careful pressure with the point of the index finger of the left hand, carried the knife through the desired depth, and gradually withdrawing it found I had made a complete section of that side, which was a little anterior to a parallel of the symphysis pubis externally. In the same manner made one upon the opposite side by changing the operating hand, and also one posteriorly. Found in the first one blunder, viz: having the thread upon the knife; and in the last section a most egregious blunder in not making this first. The merest tyro in the profession, with one ounce of reflection in his boots, should have known better. The two lateral sections left the posterior portion floating directly over the rectum, and it required no small amount of care to cut just far enough, and no farther.

The patient soon began to rally from the effects of the anodyne. The pains were stronger and more expulsive. The membranes protruding, I then without any trouble could make out the presentation, which was the first of M. Baudelocque. From this time on had the pleasure of seeing the labor progress favorably until the head entered the lower strait, when the pains began to die away, and the progress of the head seemed to be arrested from mere weariness of the uterus; fancied that it had got simply tired out like a jaded horse, and knowing that I had added nothing to the encouragement of the rest necessary by the vessels which had been divided in relieving the stricture, and the necessary effusion which must ensue, I applied the forceps (here another blunder you will say, and I not dispute) and delivered her of a large sized child (eleven pounds) which had given up the idea of vitality some time previous. The effusion of blood in the cellular tissue was much more than I had hoped it would have been; so much so that it rendered the extraction of the placenta somewhat analogous to an easy labor.

*Dec. 2d.* Found Mrs. R. this morning apparently quite comfortable; had had a somewhat restless night, but now the skin was cool, pulse 80, soft and full; had been unable to urinate; introduced a catheter and relieved the bladder. Found the bladder nearly closed from sanguineous effusion.

*Treatment.* Bled her thirty oz. in the horizontal posture; gave her sulph. morph., $\frac{1}{4}$ gr., tart. ant. one do., every four hours, and ordered warm fomentations to the vulva.

*Dec. 3d.* Appearance of the patient satisfactory; less feeling of fullness and tension of the vagina.

Continue treatment of morph. et ant. Bled her 16 oz.

From this to the tenth day treatment continued, minus venesection, and the patient gradually improving in local matters, no constitutional symptoms of any magnitude having supervened. Found suppuration had taken place to a limited extent, and opened it by way of the posterior puncture. From this time on withdrew the medication and gave her a generous unstimulating diet.

A small amount of pus was discharged, which gradually lessened from day to day, until three weeks from the time of confinement, when it ceased, and she was consigned to the benign care of the butcher.

*Remarks.* It will be seen at a glance, that Mrs. R. lost at the three bleedings, no less than 80 oz. of blood, and this, too, without any very strong constitutional symptoms. Why, by some it will be asked, was it necessary to deplete her to such an extent? The rationale, if it had one, to the treatment, I fancy is this: The anodyne kept the nervous system unconscious of the local disease, thereby continuing it as such. Prof. John Delemater, that perfect encyclopædia of medical literature, first suggested the idea to me at least, that sanguinous effusion was more rapidly absorbed when the patient was under the influence of tart. ant. Such, at least, were my impressions at the time, and such the medication from those impressions.

The query will arise in the minds of many, with regard to the anatomical relation of parts subsequent to labor. I regret to say for the edification of the profession, that with returning health also returned an unusual amount of modesty, which had been under the influence of an unusual amount of Israelitish leaven for the last few weeks, and consequently from this source could learn nothing, and all the information which her liege lord could give, was that the *peculiar* process of parturition had enhanced the properties of coition in an untold ratio. There are many things, as a little reflection will teach any one, that would render the subsequent condition of the vagina a source of much anxiety to the medical philosopher. The facts farther than stated I was unable to learn.

Some four years subsequent Mrs. R. left my ride. Two years after she again became pregnant, and died at the sixth month of gestation, in convulsions. Did not learn that an autopsy was obtained.

Should any orthopœdic surgeon flatter himself for one moment that the above operation, as simple as it may appear on paper, is in any way analogous to the division of the tendo achillis, it will require no extensive amount of practice to teach him that there is a wide difference between the division of a muscle which he can command, or cutting, as it were, in the dark with a relative change in the position of parts with each uterine pain.

East Avon, Oct., 1855.

